# Effects of Dietary Selenium Yeast Supplementation in Pregnant Cashmere Goats on the Development of Offspring Hair Follicles

**DOI:** 10.3390/ani14030477

**Published:** 2024-02-01

**Authors:** Chenxi Zhao, Yujiao Duan, Xiaogao Diao, Liwen He, Wei Zhang

**Affiliations:** 1 State Key Laboratory of Animal Nutrition and Feeding, College of Animal Science and Technology, China Agricultural University, Beijing 100193, China; 2 Sanya Institute of China Agricultural University, Sanya 572025, China

**Keywords:** maternal effect, cashmere, secondary hair follicle, oxidative stress, selenium yeast

## Abstract

**Simple Summary:**

The quality and yield of cashmere in cashmere goats are intricately linked to the morphogenesis and development of secondary hair follicles. The development of secondary hair follicles commences during the embryonic phase and is completed postnatally. Selenium can promote hair follicle development in cashmere goats, but this requires further confirmation. Our investigation revealed that maternal dietary selenium supplementation in gestation could enhance the antioxidant capacity of offspring kids, facilitate the progression of secondary hair follicle development, and subsequently improve the quality and yield of cashmere.

**Abstract:**

The objective of this study was to investigate the effects of maternal dietary selenium yeast (SY) supplementation during pregnancy on the hair follicle development of kids. Sixty pregnant Hanshan white cashmere goats were randomly divided into the con group (fed with a basal diet) and the SY group (fed with a basal diet with 0.4 mg/kg SY). SY was supplemented during the pregnancy until the birth of the kids. The growth performance, cashmere performance, hair follicle characteristics, and serum antioxidant capacity of the kids were periodically determined. The results showed that the birth weight of the kids in the SY group was significantly higher than that in the con group (*p* < 0.05), and the average weight at 15 days, 1 month, 3 months, and 5 months of age increased by 13.60%, 8.77%, 8.86%, and 3.90%, respectively (*p* > 0.05). The cashmere fineness at early birth was dramatically reduced with SY supplementation (*p* < 0.001), whereas cashmere length and production were significantly increased at 5 months of age (*p* < 0.05). Histology assays indicated that the primary hair follicles were fully developed at birth, and there was no significant difference in the number of primary hair follicles between the two groups (*p* > 0.05). The number of secondary hair follicles and the number and density of active secondary hair follicles in the SY group at 15 days were significantly higher than those in the con group (*p* < 0.05) and were increased by 11.18%, 6.18%, and 22.55% at 5 months of age, respectively (*p* > 0.05). The serum antioxidant capacity analysis revealed that the SY group had higher levels of T-AOC, SOD, CAT, and GSH-Px activities and lower levels of MDA (*p* > 0.05). These results reveal that the maternal dietary supplementation of SY in gestation can promote the morphogenesis and maturation of secondary hair follicles and increase the number and density of secondary hair follicles by enhancing the body’s antioxidant capacity, contributing to the improvement of cashmere quality and yield.

## 1. Introduction

China leads the world in cashmere goat breeding. Among the products of cashmere goats, cashmere, a rare and the finest animal fiber, is the most economically valuable product [1]. The quality of cashmere is contingent upon its fineness and length, i.e., longer and finer fibers correspond to superior quality and higher economic value. Cashmere goats are generally coated with cashmere and wool, where the fine cashmere fibers grow from secondary hair follicles, and the coarse hair fibers derive from primary hair follicles [2,3]. The morphogenesis of the secondary hair follicles of goat skin occurs during fetal life and can be fully matured until 3-6 months after birth [4,5]. Once the skin hair follicles have matured, forming a complete hair follicle structure and growing fibers, the quantity does not change throughout life. Improvements in nutrition during fetal hair follicle formation are important for increasing secondary hair follicle size, depth, and density. Maternal nutritional deficiency and oxidative stress damage during pregnancy, including a high-temperature environment and insufficient maternal oxygen supply, adversely affect the development of fetal secondary hair follicles [6,7,8]. In practice, a series of problems, such as bad environmental conditions, single forage types, and extensive feeding management, can lead to the oxidative stress of goats, resulting in an increase in free radical generation in the body and, subsequently, a change in the physiological state [7,9].

Studies have shown that the promotion of secondary hair follicle development and the augmentation of their quantity in young cashmere goats can be achieved through the administration of antioxidant substances, which effectively mitigate oxidative stress damage to the skin [10,11]. Selenium (Se) is an essential trace element that plays diverse biological roles in promoting the health and performance of animals. One of its functions is to act as a key component of glutathione peroxidase (GSH-Px), which has been reported to function as an antioxidant that removes reactive oxygen species (ROS) and protects cells from oxidative stress and DNA damage during embryonic development [12]. It was found that selenium supplementation in pregnant ewes could improve the feed utilization and body weight of kids and enhance the immune parameters and antioxidant levels of kids [13,14]. Anne et al. [15] found that maternal selenium deprivation increased DNA damage in the testicular cells of the offspring. Rock [16] and Novoselec et al. [17] reported that the dietary supplementation of organic selenium in pregnant ewes significantly improved the antioxidant status and reduced the oxidative stress of kids compared with inorganic selenium. Pregnancy is an important physiological stage during which the maternity nutritional needs increase to support fetal growth and development [18]. It is believed that maternal Se status could influence the growth and development of offspring hair follicles as nutrients, including Se, are transferred from the maternal circulation to the fetus through the placenta during pregnancy.

At present, extensive studies have focused on the effects of maternal dietary Se supplementation on the growth performance, reproduction, and immunological function of kids [12,13,14,15,16,17,18]. However, there is limited research available on the influence of maternal Se supplementation on hair follicle development and the cashmere characteristics of cashmere goat kids. Therefore, the objective of this study was to investigate the effects of maternal dietary selenium supplementation in gestation on the growth performance, cashmere properties, hair follicle development, and antioxidant statuses of kids, where sixty pregnant Hanshan white cashmere goats were randomly divided into the con group, which was fed a basal diet, and the SY group, which was fed a basal diet with 0.4 mg/kg selenium yeast (SY) during pregnancy until the birth of kids.

## 2. Materials and Methods

### 2.1. Experimental Design and Animal Management

All experimental animals were fed in the Chifeng Hanshan white cashmere goat breeding farm of the Inner Mongolia autonomous region in China, located at a longitude of 188.43° E and a latitude of 43.30° N. The trial lasted for 267 days, from 1 August 2022 to 25 April 2023. Sixty 4-year-old Hanshan white cashmere goats, which were healthy with an average body weight of 37.54 ± 1.33 kg, were selected for simultaneous estrus and breeding using artificial insemination with diluted fresh semen. Goats not returning to estrus (*n* = 40) were randomly allocated to two experimental treatments: a control group (con, *n* = 20) fed with a basal diet or the supplemented group (SY, *n* = 20) fed with a basal diet with SY (with a declared Se content of 0.4 mg/kg) and were separately housed in two sheepfolds. After a 30-day pre-feeding period, the ewes were fed from the beginning of pregnancy until delivery. The basal diets (Table 1) were formulated to meet or exceed all the nutrient requirements of cashmere goats according to the “Nutrient Requirements of Cashmere Goats (NY/T 4048-2021)” [19]. The chemical analysis of the feed was based on the official methods of analysis by the AOAC international SM (OMA) [20]. The dry matter analysis of the samples was performed by drying the samples in a forced air oven at 105 °C for 6 h. The crude protein was analyzed using the Kjeldahl method. The acid detergent fiber and neutral detergent fiber contents were determined using fiber bags (model F57; Ankom Technology, Macedon, NY, USA) and a fiber analyzer (ANKOM200 Fiber Analyzer; Ankom Technology). The calcium was analyzed via atomic absorption spectrometry. The phosphorus was analyzed via spectrophotometry. The diet was offered daily at 07:00 and 17:00 in equal allotments, roughage first and then pellets. Drinking water was freely available all the time. The selenium yeast was provided by AB MAURI Yihai Kerry Food Marketing (Shanghai) Co., Ltd., China. The concentration of elemental Se was 2000 mg/kg.

After delivering, the ewes were fed in each group according to the management of barn feeding. The eves and offspring kids were fed separately in the barn. The kids suckled three times a day (7:00 a.m.~8:00 a.m., 13:00 p.m.~14:00 p.m., and 19:00 p.m.~20:00 p.m.). The kids were fed guinea grass and pellets at 15 days of age and weaned at 3 months of age. The kids were fed freely on guinea grass and quantitatively on pellets, while drinking water was freely available all the time. The pellet was purchased from Wanjia Feed Co., Ltd. (Beijing, China). The basic nutritional value content analysis according to OMA is shown in Table 2 [20].

The management of the animals and procedures used in this study were carried out following the guidelines of the Animal Care and Use Committee of China Agricultural University (Beijing, China).

### 2.2. Live Weight and Collection of Samples

After delivery, 16 kids were randomly selected from each of the two groups as experimental animals. Each group included 8 single kids (4 males and 4 females) and 8 twin kids (5 males and 3 females) for data recording and sample collection. The specific indicators were as follows:

The regular fasting weight of each kid in the two groups was recorded at the ages of 1 day, 15 days, 3 months (weaning), and 5 months.

Cashmere fiber samples within an area of 5 × 5 cm were shorn close to the skin on the left mid-side flank region of each cashmere goat kid for the measurement of fiber staple length and diameter at 15 days and 5 months of age. The weight of total cashmere production for each kid was recorded after combing.

Two skin samples were collected (at ages of 15 days and 5 months) at the posterior edge of the left or right scapula and the upper 1/3 between the dorsal and ventral midlines. After alcohol disinfection, a ring skin sampler (with a diameter of 1 cm) was used to gently press the skin to collect samples, and then Yunnan Baiyao was applied at the sampling site. Collected skin specimens were placed flat on an embedding case, soaked in 4% paraformaldehyde solution for 24 h, and dehydrated in a series of graded ethanol solutions for paraffin embedding and section preparation.

Approximately 5 mL vein blood samples of each kid were collected in the morning on each skin biopsy sampling day and then centrifuged at 1500 rpm/min for 10 min at 4 °C to separate the serum, which was collected into a small cryotube (2 mL) and stored at −20 °C until analysis.

### 2.3. Cashmere Fiber Measurements

The stretch length of the cashmere fibers was measured with a steel ruler by dipping a finger in a small amount of water and gently stroking the wool fiber until all bends disappeared. The total measured number of each cashmere fiber sample was 100. The cashmere diameter was measured via an optical microscopic projection method using an automated analyzer (Optic Fiber Diameter Analyzer, CU-6, Beijing United Vision Technical Company, Beijing, China). Each cashmere sample was determined to have a mean value of 200 fibers.

### 2.4. Determination of the Parameters Related to the Hair Follicle Population

Images of the hair follicles were taken with a microscope camera (Leica ICC 50 W, Leica, Wetzlar, Germany). A total of 10 microscopic fields were obtained from the sections of each sample used for hair follicle counting. The hair follicle index included the total number of primary, secondary, and mature or active secondary hair follicles, primary hair follicle density (PFD), secondary hair follicle density (SFD), mature or active secondary hair follicle density (MSFD), the ratio of total secondary hair follicles to total primary hair follicles (S:P), and the ratio of total mature or active secondary hair follicles to total primary hair follicles (Sf:P). The procedures for determining the cashmere traits and hair follicle indexes are found in Duan and Yang et al. [21,22].

### 2.5. Estimation Activities of the Antioxidative Enzymes

The total superoxide dismutase (T-SOD) activity was measured using the hydroxylamine method (sensitivity level: 5.0 U/mL). The measurement of catalase (CAT) activity was performed using the ammonium molybdate method (sensitivity level: 0.2 U/mL), and the GSH-Px activity was measured via colorimetry (sensitivity level: 20 U). The total antioxidative capacity (T-AOC) was evaluated using the FRAP method (sensitivity level: 0.2 U/mL). The malondialdehyde (MDA) content was determined using the TBA method (sensitivity level: 0.5 rmol/mL). All of the enzyme assays were conducted with kits (Nanjing Jiancheng Bioengineering Institute, Nanjing, China), and the procedures were performed strictly according to the manufacturer’s instructions.

### 2.6. Statistical Analyses

All analyses were performed using SPSS 27.0 and GraphPad 9.0. The results were expressed as the mean value ± SEM. The data on the cashmere performances and parameters related to hair follicles were compared between the kids in the con and SY groups with Student’s *t*-test. The difference was considered significant at *p* < 0.05.

## 3. Results

### 3.1. Growth Performance

**Figure 1:**
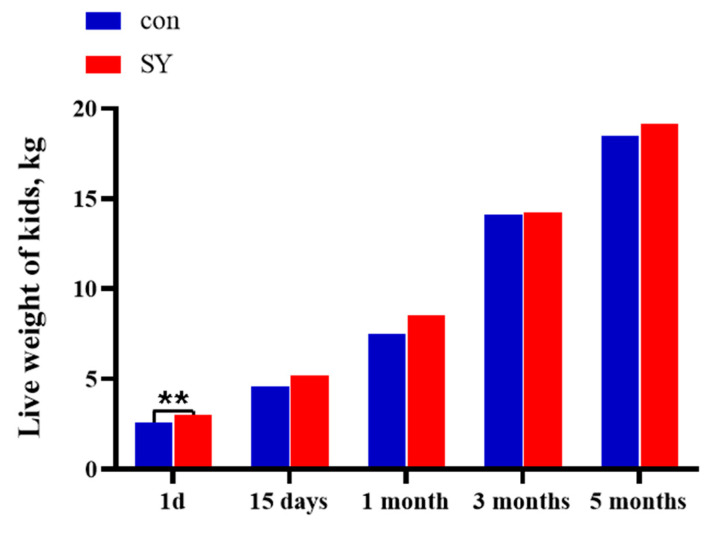
Effects of maternal dietary selenium yeast on the growth performance of kids. The symbol indicates the level of significance of the differences (** *p* < 0.01, SY vs. con).

Figure 1 shows the live weight of goat kids in the con and SY groups at different time points. The weight of the kids in both groups increased with age. The birth weight of the kids in the SY group was significantly higher than that in the con group (*p* < 0.01). Compared to that in the con group, the body weight of the kids in the SY group at 15 days, 1 month, 3 months (weaning), and 5 months (combing) of age represented an increase of 13.60%, 8.77%, 8.86%, and 3.90%, respectively (*p* > 0.05).

### 3.2. Cashmere Yield and Fiber Quality

**Figure 2:**
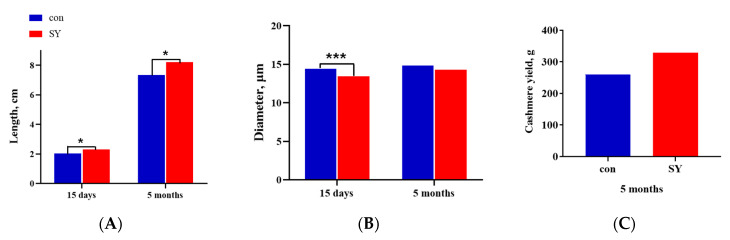
Effects of maternal dietary selenium yeast on the cashmere production performance of kids. (**A**) Cashmere fiber length of the SY group and con group goat kids at different times. (**B**) Cashmere fiber diameter of the SY group and con group goat kids at different times. (**C**) Cashmere yield of the SY group and con group goat kids at the first shearing. Different symbols indicate the level of significance of the differences (* *p* < 0.05, *** *p* < 0.001, SY vs. con).

Figure 2 shows the effects of SY supplementation during pregnancy on the yield and quality of the cashmere fiber of the offspring. The average staple length of the cashmere fiber of the kids in the SY group at 15 days represented a significant increase in comparison with that of the con group (2.31 ± 0.10 cm vs. 2.06 ± 0.06 cm, *p* < 0.05; Figure 2A) and increased remarkably at 5 months (8.21 ± 0.24 cm vs. 7.35 ± 0.22 cm, *p* < 0.05; Figure 2A).

The cashmere fiber diameter of the kids in the SY group at 15 days was significantly finer than that in the con group (13.40 ± 0.14 μm vs. 14.40 ± 0.14 μm, *p* < 0.001; Figure 2B) and reduced by 0.57 μm in comparison with that of the con group at the age of 5 months (14.29 ± 0.13 μm vs. 14.86 ± 0.37 μm, *p* > 0.05; Figure 2B). The average cashmere yield of the kids in the SY group was significantly higher than that of the con group at first combing (694.8 ± 14.2 g vs. 620.2 ± 53.1 g, *p* < 0.05; Figure 2C).

### 3.3. Primary and Secondary Hair Follicles

**Figure 3:**
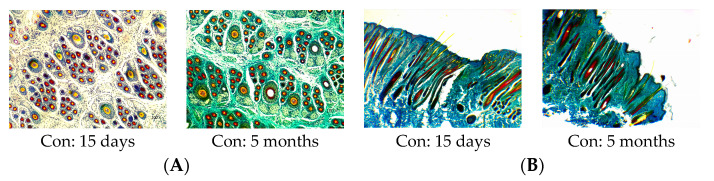

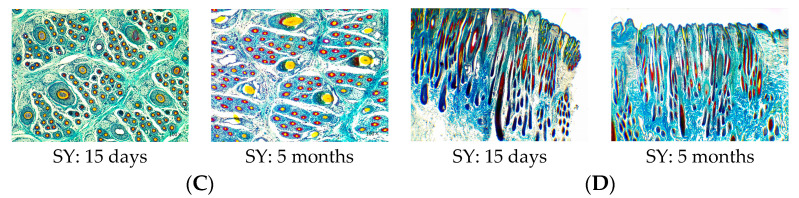
Effects of maternal dietary selenium yeast on the morphological changes of the hair follicles of kids at different time points. (**A**) Cross-section of the hair follicles in the con group, 100×. (**B**) Longitudinal section of the hair follicles in the con group, 100×. (**C**) Cross-section of the hair follicles in the SY group, 100×. (**D**) Longitudinal section of the hair follicles in the SY group, 100×.

Figure 3 shows the cross and longitudinal sections of the hair follicles on the 15th day and 5th month. As seen in the cross-section, most primary hair follicles completed development on the 15th day, while the secondary hair follicles were immature and few in number. The hair follicle morphology shows purple cell clusters in the differentiation stage. At 5 months of age, almost all secondary hair follicles were matured. The total secondary hair follicles and active secondary hair follicles in the SY group were significantly higher than those in the con group. The longitudinal section shows that the number of cashmere fibers at the skin level was more than that in the con group, and the fineness was thinner.

Table 3 provides an intuitive impression of the effects of maternal selenium treatment during pregnancy on the hair follicle development of kids. The total number of primary hair follicles of cashmere goats in the SY group and con group were basically unchanged, which was not affected by the sampling time (*p* > 0.05). The PFD decreased with age in both groups, but there was no significant difference (*p* > 0.05). For the secondary hair follicles, SY supplementation changed the parameters. The total number of secondary hair follicles at 15 days of age in the SY group was significantly higher than that of the con group (*p* < 0.05), and the total number of secondary hair follicles at 5 months of age was 11.18% higher than that of the con group (*p* > 0.05). Despite no significant effect on the SFD, SY supplementation increased the SFD by 16.77% and 11.18% than that of the con group, respectively, at two time points (*p* > 0.05). Additionally, the MSFD and total active secondary hair follicles were significantly different between the two groups at 15 days of age (*p* < 0.01) and increased by 6.13% and 22.55% in the SY group compared with the con group at 5 months of age, respectively (*p* > 0.05). Compared with the control group, the S:P of kids aged 15 days and 5 months in the SY group was increased by 10.92% and 3.83%, respectively (*p* > 0.05). The addition of SY during pregnancy significantly increased the Sf:P of kids at 15 days of age (*p* < 0.05), with a 3.22% increase observed in the SY group at 5 months of age (*p* > 0.05).

### 3.4. Antioxidas, Lipid Peroxidation, and Total Antioxidant Capacity

**Figure 4:**
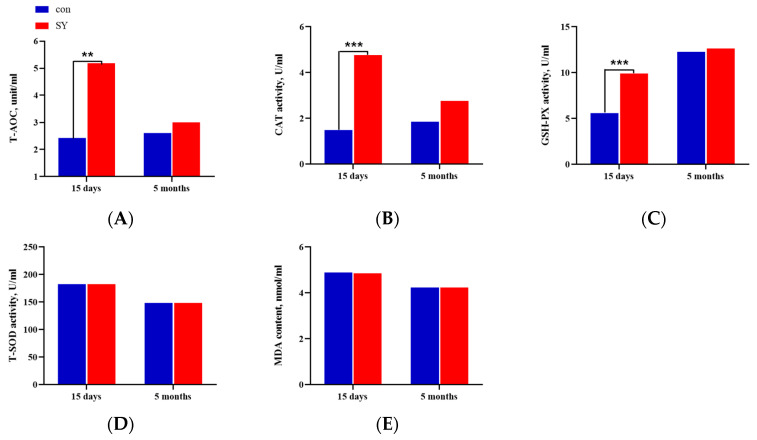
Effects of maternal dietary selenium yeast on antioxidase activities, lipid peroxidation, and total antioxidant capacity in the serum of kids at different time points. (**A**) Serum T-AOC of the SY group and con group goat kids at different times. (**B**) Serum CAT activities of the SY group and con group goat kids at different times. (**C**) Serum GSH-Px activities of the SY group and con group goat kids at different times. (**D**) Serum T-SOD activities of the SY group and con group goat kids at different times. (**E**) Serum MDA content of the SY group and con group goat kids at different times. Different symbols indicate the level of significance of the differences. (** *p* < 0.01, *** *p* < 0.001, SY vs. con).

The serum antioxidant statuses of the kids in different groups are shown in Figure 4. At 15 days of age, the serum T-AOC of the kids in the SY group was significantly higher than that in the con group (*p* < 0.01; Figure 4A). Similarly, the CAT and GSH-Px activities were significantly different between the two groups (*p* < 0.001; Figure 4B,C). However, there were no differences in the total SOD activity and MDA content in the serum between the two groups at this stage (*p* > 0.05; Figure 4D,E). At 5 months of age, the activities of the antioxidant enzymes decreased compared with those at 15 days of age, but the comprehensive antioxidant capacity of the kids in the SY group was still better than that of the con group (*p* > 0.05). The activities of T-AOC, CAT, GSH-Px, and total SOD in the serum of the kids in the SY group were higher than those in the con group, and the content of MDA was lower than that in the con group (*p* > 0.05).

## 4. Discussion

Maternal nutritional status is one of the external factors that determines the nutrient distribution and, ultimately, affects the growth, development, and function of major fetal organ systems [23]. In mammals, Se is transferred to the fetus or offspring through the placenta and mammary glands. Recent studies have shown that selenium supplementation in the maternal diet is important for the development of offspring [13,14,15,16]. In this study, the effects of the supplementation of SY during pregnancy on the development of hair follicles in newborn cashmere goats were investigated. The cashmere production performance of kids in the SY group was better than that of the con group, and the number of secondary hair follicles and active secondary hair follicles were also greatly increased, which were manifested by the increased length of cashmere and the finer diameter of the cashmere fibers.

Selenium supplementation during the pregnancy of ewe can promote placenta growth, affect maternal mammary gland development, and improve the production and composition of milk, thus promoting the growth and development of fetuses and kids [24]. In this study, selenium supplementation in pregnant ewes increased the average body weight of offspring kids, which was consistent with the results of Camacho [25] and Hassan et al. [26], who found that selenium supplementation during pregnancy effectively led to an improvement in the birth and weaning weight of kids. Cappai et al. found a difference in the fluid balance and distribution of the gestating and lactating goats when single or twin kids were raised [27]. When selecting experimental kids, we considered and balanced the effects of pregnancy differences and gender on nutrient intake and growth performance and concluded that the average body weight in the two groups was representative to a certain extent. Hanshan white cashmere goat coat is composed of wool and cashmere, corresponding to the primary and secondary hair follicles, respectively. The skin hair follicles are regularly distributed in the form of hair follicle groups, which are surrounded by dense connective tissue. The hair follicle group in the skin of Hanshan white cashmere goats is mainly a trichotomous group; that is, a hair follicle group consisting of three primary hair follicles and several secondary hair follicles. The diameter of the primary hair follicles is relatively large, and the hairballs are located in the deep skin, while the diameter of the secondary hair follicles is thinner, and the hairballs are located in the superficial layer of the skin. With age, secondary hair follicles grow to the root of the primary hair follicles, indicating that the occurrence and development of secondary hair follicles in Hanshan white cashmere goats are similar to other mammals [28,29,30]. Cashmere fiber diameter and cashmere yield are important indicators to measure the economic value of cashmere [5]. This study showed that selenium supplementation in pregnant ewes could increase the length of cashmere, reduce the diameter of cashmere, and increase the production of cashmere at the first combing stage (5 months of age) in their goat kids. Especially in the early period after birth, the cashmere length and diameter of the kids in the SY group significantly increased. These results indicate that the maternal selenium effect could significantly affect the fleece production performance of newborn kids. However, the beneficial effects of selenium might not last as long as kids grow independently of their mothers. Moreover, Pallotti et al. found a negative correlation between cashmere yield and cashmere diameter [31]. Wang et al. believed that cashmere yield was positively correlated with body weight and cashmere length [32]. The correlations among the character parameters of cashmere in this study confirmed the conclusions of previous studies. According to the “Chinese Cashmere Quality Standard” [33], we believe that the cashmere textile value of the kids in the SY group is better than that of the con group. Therefore, selenium supplementation for pregnant ewes could improve the cashmere quality and economic value of offspring kids at first combing.

The development of secondary hair follicles depends on the interaction between epidermal keratinocytes and subdermal mesenchymal cells [34,35,36]. Selenoproteins are essential for the normal function of keratinocytes and skin development [37]. In this study, the number and density of secondary hair follicles and mature secondary hair follicles of kids at early birth in the SY group were significantly higher than those in the con group. At 5 months of age, the number of secondary hair follicles and mature secondary hair follicles in the SY group increased by 11.18% and 6.13% compared with the con group, respectively. These results indicate that selenium supplementation in pregnant ewes could promote the development and maturation of secondary hair follicles in offspring kids, which is consistent with the results of Wu et al. [10]. Most of the primary hair follicles of cashmere goats are developed before birth, and the development of primary hair follicles is almost unaffected by nutrition and environmental factors [38,39]. In the present study, the total primary hair follicles of cashmere goats did not change with an increase in age, indicating that the primary hair follicles were fully developed at birth. For adult cashmere goats, it is difficult to reach the skin S:P value at birth, indicating that the secondary hair follicles formed in the fetal period significantly subside during growth and development. We found that the total secondary hair follicles of 5-month-old cashmere goats were lower than those of 15-day-old cashmere goats, indicating the existence of apoptosis during the maturation of the secondary hair follicles of cashmere goats, which was consistent with the observation of secondary hair follicle apoptosis during the maturation of Inner Mongolia cashmere goats [11]. During the whole experimental period, the SFD, MSFD, and S:P of the kids in the SY group were higher than those in the con group, especially in the early stage after birth, indicating that the selenium supplementation of pregnant ewes could inhibit the apoptosis of secondary hair follicles and promote the development and maturity of secondary hair follicles. There was a positive correlation between cashmere yield and secondary hair follicle density and a negative correlation between cashmere diameter and secondary hair follicle density. In this study, the SFD and metabolic activity of secondary hair follicles were increased in the SY group, which achieved the purpose of simultaneously reducing the cashmere diameter and increasing the cashmere yield. The results showed that selenium supplementation for pregnant ewe was an effective method to increase the number of secondary hair follicles, improve the quality of cashmere, and increase the yield of cashmere.

During secondary hair follicle development, reactive oxygen species and their associated oxidative stress cause damage to cells, including proteins and DNA, impairing the self-renewal, differentiation, and proliferation ability of mesenchymal stem cells (MSCs) and epidermal stem cells and inducing cell apoptosis, thus inhibiting secondary hair follicle development [40,41,42]. A previous study showed that antioxidant supplementation could improve antioxidant capacity and promote secondary hair follicle development in the skin. After the dietary supplementation of coenzyme Q10, T-AOC and the hair follicle density of gonadectomy mice were increased [43]. A previous study by our research group found that the number and density of secondary hair follicles and the antioxidant capacity of cashmere goats significantly increased after melatonin administration [11,38]. Based on the above conclusions, we measured the activity of serum antioxidant enzymes to investigate whether the improvement in secondary hair follicle parameters with selenium yeast was mediated by the antioxidant capacity of the body. T-AOC is an important index, indicating the gross antioxidant capacity of an animal’s body, and CAT and GSH-Px activities reflect the free radical scavenging ability of an animal’s body [44]. The results showed that selenium supplementation in pregnant ewes could increase the activities of CAT, GSH-Px, and T-AOC of offspring kids, decrease MDA content, and reduce oxidative stress damage, consistent with the results of Ibrahim et al. [45], and Y. et al. [46], which were that selenium could improve the antioxidant capacity of the body. At the same time, the MSFD, total secondary hair follicles, and total active secondary hair follicles of the kids in the SY group were significantly higher than those in the con group, indicating that selenium might affect the development of secondary hair follicles by improving the body’s antioxidant capacity and reducing oxidative stress. It is consistent with the results reported by Wu et al., which were that selenium supplementation in pregnant ewes during the period from 30 days before gestation to 110 days at fetus promoted the formation and maturation of fetal secondary hair follicles [10]. At 5 months of age, there were no significant differences in the antioxidant enzyme activity and antioxidant capacity between the two groups, but these parameters were numerically higher in the SY group. In fact, the kids no longer received an external selenium supply after weaning at 3 months of age, indicating that the maternal effect no longer significantly affected the independent growth and development of kids after the maternal transfer of selenium was metabolized in vivo. T-SOD produces a small amount of molecular oxygen or hydrogen peroxide while reducing superoxide anions, but its products can be offset by CAT or GSH-Px, thereby reducing the damage of free radicals to cells [47]. The results of this study showed that the serum T-SOD activity of SY-group kids was not significantly increased, which was inconsistent with the study results of Shi et al. [48]. However, Mousaie et al. found that the SOD activity of lambs was not significantly increased after supplementation with selenium yeast [14]. In this regard, it is speculated that the results may be biased due to different goat breeds, ages, and feeding conditions. Based on the above results, it is believed that maternal selenium supplementation during pregnancy may promote the development of secondary hair follicles in cashmere goats by improving the antioxidant capacity of offspring kids and reducing oxidative stress.

## 5. Conclusions

This study provides potent evidence that maternal dietary selenium yeast supplementation during the pregnancy of cashmere goats can improve the growth performance of offspring kids, especially early kids, and improve the cashmere quality and yield of kids. Moreover, for offspring kids, maternal dietary selenium yeast supplementation can promote the morphogenesis and maturation of secondary hair follicles by enhancing antioxidant capacity and reducing oxidative stress, and subsequently increasing the number and density of secondary hair follicles.

## Figures and Tables

**Table 1:** Ingredients and chemical compositions of the maternal basal diets.

Item	Early Gestation	Late Gestation	Lactation
Ingredient (%)			
Guinea grass	70.00	65	65
Corn	11.67	14.63	13.00
Wheat bran	10.89	12.35	12.41
Soybean meal	5.93	6.65	8.11
NH_4_H_2_PO_4_	0.32	0.26	0.35
NaCl	1.00	1.00	1.00
Trace mineral mix ^a^	0.12	0.12	0.12
Chemical composition ^b^			
Digestible energy, MJ/kg	10.58	10.94	11.51
Dry matter	91.40	91.35	91.78
Crude protein	10.03	10.85	11.07
Acid detergent fiber	26.35	25.02	25.17
Neutral detergent fiber	53.49	51.46	51.20
Calcium	0.58	0.55	0.55
Phosphorus	0.17	0.17	0.18

^a^ Provided per kilogram of the gestational diet: 40 mg of Zn as ZnSO_4_·H_2_O, 20 mg of Cu as CuSO_4_·5H_2_O, 0.1 mg of Co as CoCl_2_·6H_2_O, and 5000 IU of vitamin A. ^b^ Analyzed values except for DE.

**Table 2:** Chemical compositions of the offspring’s basal diets.

Chemical Composition ^a^	Content (%)
Digestible energy, MJ/kg	11.45
Dry matter	89.10
Crude protein	18.89
Acid detergent fiber	18.25
Neutral detergent fiber	37.22
Calcium	0.55
Phosphorus	0.32

^a^ Analyzed values except for DE.

**Table 3:** The hair follicle trial at different time points.

Age	Item	Group		*p*-Value
		Con	Sy	
15 days	PF, *n*/million	36.27 ± 1.76	40.75 ± 2.02	0.112
	PFD, *n*/mm^2^	7.54 ± 0.75	7.66 ± 0.38	0.886
	SF, *n*/million	388.46 ± 19.49	434.07 ± 22.60	0.025
	SFD, *n*/mm^2^	73.13 ± 3.66	77.60 ± 3.89	0.104
	MSFs, *n*/million	325.55 ± 18.49	391.38 ± 20.26	0.025
	MSFD, *n*/mm^2^	61.30 ± 3.48	73.69 ± 3.82	0.025
	S:P	10.24 ± 0.92	11.36 ± 1.00	0.424
	Sf:P	7.74 ± 0.52	10.20 ± 0.81	0.025
5 months	PF, *n*/million	38.57 ± 2.92	39.33 ± 2.13	0.832
	PFD, *n*/mm^2^	4.36 ± 0.39	4.64 ± 0.46	0.662
	SF, *n*/million	214.61 ± 23.85	238.60 ± 20.20	0.453
	SFD, *n*/mm^2^	40.41 ± 4.49	44.93 ± 3.80	0.453
	MSFs, *n*/million	203.33 ± 21.34	215.79 ± 11.40	0.618
	MSFD, *n*/mm^2^	34.97 ± 4.74	42.86 ± 3.77	0.208
	S:P	5.95 ± 0.97	6.18 ± 0.51	0.827
	Sf:P	5.14 ± 0.90	5.89 ± 0.49	0.842

PF = total primary hair follicles, SF = total secondary hair follicles, MSFs = mature or active secondary hair follicles.

## Data Availability

The data used to support the findings of this study will not be publicly available as we will be conducting further research.
